# Biocompatibility and Bioimaging Potential of Fruit-Based Carbon Dots

**DOI:** 10.3390/nano9020199

**Published:** 2019-02-03

**Authors:** Cindy Dias, Nagamalai Vasimalai, Marisa P. Sárria, Ivone Pinheiro, Vânia Vilas-Boas, João Peixoto, Begoña Espiña

**Affiliations:** 1 INL—International Iberian Nanotechnology Laboratory, 4715-330 Braga, Portugal; cindydias93@gmail.com (C.D.); vasimalai.gri@gmail.com (N.V.); ivone.pinheiro@inl.int (I.P.); vfevilasboas@gmail.com (V.V.-B.); 2 CEB—Centre of Biological Engineering, University of Minho, 4720-057 Braga, Portugal; jmp@deb.uminho.pt; 3 Department of Chemistry, B.S. Abdur Rahman Crescent Institute of Science and Technology, Vandalur, Chennai-600048, India; 4 UCIBIO-REQUIMTE, Laboratory of Toxicology, Biological Sciences Department, Faculty of Pharmacy, University of Porto, Rua de Jorge Viterbo Ferreira, 228, 4050–313 Porto, Portugal

**Keywords:** carbon dots, bioimaging, zebrafish embryotoxicity, cytotoxicity, biocompatibility

## Abstract

Photo-luminescent carbon dots (CD) have become promising nanomaterials and their synthesis from natural products has attracted attention by the possibility of making the most of affordable, sustainable and, readily-available carbon sources. Here, we report on the synthesis, characterization and bioimaging potential of CDs produced from diverse extensively produced fruits: kiwi, avocado and pear. The in vitro cytotoxicity and anticancer potential of those CDs were assessed by comparing human epithelial cells from normal adult kidney and colorectal adenocarcinoma cells. In vivo toxicity was evaluated using zebrafish embryos given their peculiar embryogenesis, with transparent embryos developing ex-utero, allowing a real-time analysis. In vitro and in vivo experiments revealed that the synthesized CD presented toxicity only at concentrations of ≥1.5 mg mL^−1^. Kiwi CD exhibited the highest toxicity to both cells lines and zebrafish embryos, presenting lower LD_50_ values. Interestingly, despite inducing lower cytotoxicity in normal cells than the other CDs, black pepper CDs resulted in higher toxicity in vivo. The bio-distribution of CD in zebrafish embryos upon uptake was investigated using fluorescence microscopy. We observed a higher accumulation of CD in the eye and yolk sac, avocado CD being the ones more retained, indicating their potential usefulness in bio-imaging applications. This study shows the action of fruit-based CDs from kiwi, avocado and pear. However the compounds present in these fruit-based CDs and their mechanism of action as a bioimaging agent need to be further explored.

## 1. Introduction

Semiconductor quantum dots (q-dots) hold much attention for their various potential applications in optical bioimaging and biomedical devices among others [1]. Because of their unique photoelectric proprieties, q-dots are generally considered as an alternative to conventional organic dyes [2]. However, the most traditional q-dots contain heavy metal elements, which raise significant concerns about the impact of using these nanomaterials in biological systems due to their potential human and environmental toxicity [3]. Carbon dots (CD) are a novel class of nanomaterials that have lately received a high degree of attention and investigation as they present the same major advantageous characteristics of semiconductor q-dots, such as high photostability and tunable emission [4]. However, compared with semiconductor q-dots, CDs exhibit excellent aqueous solubility, high biocompatibility and are cheaper to produce [5]. Another important fact is that CDs seem to be more environmentally friendly and could be safer for biological use [6]. Nowadays, multiple green chemistry techniques are described to obtain CD, as well as different carbon sources for them [7,8]. Recently, among the natural carbon sources that can be used for the synthesis of CD, food products such as fruits have been explored [9]. A recent work is recommended for an extensive revision of the reported literature on CD produced from natural sources [10].

Globally, the annual production is around 24, 4 and 6 millions of tons for pear, avocado and kiwi, respectively [11]. Kiwi and avocado are among the crops more affected by spoilage and are extensively culled out in harvesting, raising an extremely significant loss of up to 40% at the production and commercialization level [12].

Additionally, phenolic and polyphenolic compounds, which present antioxidant properties, are extensively present in fruits, making them an attractive carbon source for CDs synthesis. CDs have been previously synthesized from grapes, lemon, lychees and other fruits [13,14,15], but not from kiwi or avocado, which present a high amount of polyphenolic compounds, among others, and their production on the global scale is high [16,17,18,19,20,21].

Therefore, we selected kiwi, pear and avocado as carbon sources for the synthesis of CDs, according to their potential added value, mainly due to their richness in phenolic and polyphenolic compounds and their global level of production and loss. This selection has the intention of helping in the food products’ waste valorization, increasing the sustainability of the agriculture activities and diminishing the carbon footprint. To the best of our knowledge, this is the first time that kiwi and avocado CDs are reported in the literature.

After synthesis, a rigorous assessment of the toxicological profile of CDs, both in vitro and in vivo is of utmost importance, so that they can be validated and further applied.

In vitro cytotoxicity testing is generally implemented to obtain a first screening of nanoparticles’ toxicity [22,23]. Still, it does not replace the biological complexity represented by in vivo models. Issues such as route of administration, biodistribution, biodegradability, long-term disposition, and induction of developmental defects are major advantages of in vivo nanotoxicity assessment, and cannot be properly addressed using in vitro experimental setups [22,24]. Therefore, comprehensible and consistent in vivo models are required to assess the toxicity of nanomaterials and, thereby bridge in vitro cell models and small mammalian models.

Zebrafish, *Danio rerio* (Hamilton 1822), is a model organism that has rapidly gained interest in biomedical research [25] because of its high degree of homology to the human genome (approximately 70%) and human metabolism, rapid *ex-utero* embryonic development, and short generation time [26,27,28,29]. These characteristics, combined with their high fecundity (zebrafish females lay around 200 to 300 eggs every 5 to 7 days) and the optical transparency of the newborns throughout embryogenesis, allowing real-time screening of the test conditions and inexpensive investment to raise them, define this species’ unique advantages compared to other vertebrate models such as mice and rats [30,31,32,33,34]. The zebrafish embryo toxicity (ZET) assay is relatively well established in the environmental sciences to assess both toxic effects and the development defects resulting from environmental exposure [10].

In the present study, three different CDs were synthesized from three of the most extensively produced fruits: kiwi, pear and avocado fruits, by one green-pot hydrothermal method, and investigated for their toxicity and biodistribution using both in vitro and in vivo models. Taking into account a previous work with spices and ginger CD [35], where their anticancer potential was demonstrated, novel fruit-based CD were also screened for their anticancer potential by studying their differential toxicity against normal and cancer human epithelial cell lines. Moreover, due to the fluorescence of CD, the potential bioimaging application of fruit-based CD was further investigated through fluorescence microscopy

## 2. Materials and Methods

### 2.1. Chemicals and Reagents

4-(2-hydroxyethyl) pipearzine^−1^-ethanesulfonic acid (HEPES), 2-(N-morpholino) ethanesulfonic acid (MES) were obtained from Sigma-Aldrich (Madrid, Spain) and phosphate-buffered saline (PBS) from GIBCO Invitrogen (Barcelona, Spain). Ultrapure water (18.2 MΩ cm at 25 °C) was used for the buffers’ preparation. Antibiotic mixture of penicillin/streptomycin (10,000 U mL^−1^/10,000 μg mL^−1^) and Amphotericin B (Fungizone-250 μg/mL) were purchased from GIBCO Invitrogen (Barcelona, Spain). Dulbecco’s Modified Eagle’s medium (DMEM) with a high glucose content was acquired from Sigma-Aldrich (Madrid, Spain). Non-Essential Amino Acids Solution 100× (MEM), Dulbecco’s Modified Eagle’s medium with nutrient mixture F^−1^2 (DMEM/F^−1^2), GlutaMAX-I™, trypsin 0.25%-EDTA, human transferrin (4 mg mL^−1^) and PrestoBlue™ cell viability reagent were acquired from GIBCO Invitrogen (Barcelona, Spain). Fetal bovine serum (FBS) was obtained from HyClone GE Healthcare (Carnaxide, Portugal).

The immortalized proximal tubule epithelial cell line derived from normal adult human kidney HK-2 (ATTC^®^CRL-2190™) and the human epithelial colorectal adenocarcinoma Caco-2 (ATTC^®^HTB-37™) cell lines were obtained from ATCC (LGC Standards S.L.U., Barcelona, Spain).

Polyethylene glycol p-(1,1,3,3-tetramethylbutyl)-phenyl ether (Triton X^−1^00), 3-Aminobenzoic Acid Ethyl Ester Methanesulfonate (Tricaine), paraformaldehyde (PFA) and Methanol were obtained from Sigma-Aldrich (Madrid, Spain) and agarose from Fisher BioReagent. These chemicals were used for fixation of the zebrafish embryos.

All other chemicals not listed in this section were of the highest purity grade commercially available.

### 2.2. Synthesis of CD

All CD were synthesized by the hydrothermal method. Briefly, 20 mL of each fruit juice (kiwi, avocado, and pear) were sonicated for 15 min at 80 kHz, 25% ultrasonication power at 30 °C temperature. Afterwards, the mixture was stirred for 5 min followed by a hydrothermal treatment at 200 °C for 12 h using Teflon-coated autoclave tubes. Then, the resultant black carbonized solution was cooled down to room temperature and filtered through 0.22 µm cellulose ester-mixed Whatman filter paper to remove the large particles. The obtained brownish-yellow filtrate solution was dialyzed for 6 h in 1 L Milli-Q water using a dialysis membrane with 3.5 kDa MWCO, replacing the water every 30 min.

For citrate CD synthesis, 2.0 g of citric acid were dissolved in 20 mL of 1 mol L^−1^ phosphate buffer at pH = 7.2. Twenty millilitres of this solution was used for the hydrothermal process and subsequent purification steps described above. Black pepper CDs were synthesized as follows: 2.0 g of black pepper powder was diluted in 10 mL of Milli-Q water and sonicated for 30 min at 80 kHz and 25% sonication power at 30 °C. Afterwards, the mixture was stirred for 15 min followed by a hydrothermal treatment at 200 °C for 12 h using Teflon-coated autoclave tubes. The resultant black carbonized solution was cooled down to room temperature. The same purification process was carried out as in Reference [36]. Finally, 1 mL of the purified CD was aliquoted and dried at 100 °C until a stable weight was obtained. Subsequently, the concentration of CD was calculated by the weight loss method.

### 2.3. Characterization of the CDs

Fluorescence spectra were measured using a Horiba Scientific Fluoromax-4 Instrument (Horiba Scientific, Piscataway, NJ, USA), equipped with a xenon discharge lamp, and a 1 cm quartz cell at room temperature. For all the fluorescence measurements, excitation and emission slit widths were kept at 5 nm. UV-visible measurements were performed on a Shimadzu UV-2550 UV-Vis spectrophotometer (Shimadzu Corporation, Tokyo, Japan). The concentration of all CDs was 2 mg mL^−1^ for fluorescence and absorption measurements. Transmission electron microscopy (TEM) experiments were carried out with a JEOL-2100 transmission electron microscope (JEOL Ltd., Tokyo, Japan) working at 200 keV. For TEM sample preparation, the CDs were placed onto formvar-carbon-coated copper TEM grids with 400 mesh (Agar Scientific, Essex, UK) and dried.

### 2.4. In Vitro Cytotoxicity Evaluation

#### 2.4.1. Cell Lines and Culture Conditions

Epithelial human kidney cells HK-2 (ATTC^®^CRL-2190™) and epithelial human colorectal adenocarcinoma cells Caco-2 (ATTC^®^HTB-37™) were used to study the in vitro cytotoxic profile of the fruit-based CD. HK-2 cells were cultured and maintained in DMEM/F12 medium supplemented with FBS (10%), penicillin/streptomycin (1%), fungizone (2.5 μg/mL) and human transferrin (5 μg/mL). Caco-2 cells were cultured and maintained in DMEM supplemented with FBS (20%), penicillin/streptomycin (1%) and non-essential amino acids (1%). HK-2 cell line (from passages 10 to 33) and Caco-2 cell line (from passages 18 to 42) were kept in a humidified incubator with 5% CO_2_, at 37 °C and sub-cultured every 3 to 4 days in order to maintain sub-confluence.

#### 2.4.2. Cytotoxicity Tests

HEPES E3 buffer (i.e., HEPES 15 mmol/L with 5 mmol/L NaCl, 0.17 mmol/L KCl, 0.33 mmol/L CaCl_2_ and 0.33 mmol/L MgSO_4_ pH = 7.2, prepared in ultrapure water) was used to prepare all the CDs stock solutions. The solutions were sterilized by filtration through a 0.22 μm pore size filter and diluted in the respective cell medium to prepare the different test concentrations. Cells were seeded in 96-well plates at an initial density of 1 × 10^5^ cells/mL, and left overnight for adherence at 5% CO_2_ and 37 °C. The cellular density and viability were determined by counting the cells in a Neubauer chamber using Trypan Blue dead cells exclusion. After 24 h, the medium was replaced and both cell lines were exposed to serial dilution concentrations of fruit-based CD, by duplicate, for 48 h and 72 h. The following controls were considered: negative (viability) control, i.e., cells incubated with cell culture media; positive (death) control, i.e., cells incubated with 30% (v:v) dimethyl sulfoxide (DMSO); vehicle control, i.e., cells incubated with 45 μL of HEPES E3 buffer and 55 μL of cell culture media. The referred volume of HEPES E3 simulates the highest concentration of buffer used when preparing the test concentrations of the CD.

Cytotoxicity was evaluated using the PrestoBlue^®^ (PB) cell viability assay. Briefly, after 48 h or 72 h of exposure to the different concentrations of the fruit-based CD, PB was added to each well (at a 1:10 dilution) and the microplate was incubated for 1 h at 37 °C.

Fluorescence due to the reduction of the dye by the cells’ metabolism, was registered at 560 and 590 nm excitation and emission wavelengths, respectively, using a Synergy H1 microplate reader (BioTek^®^). Auto-fluorescence of the fruit-based CD in the different cell culture media was analyzed to avoid misleading results (Table S1).

No significant interference of CD’s with PB’s fluorescence was observed (data not shown).

### 2.5. In Vivo Nanotoxicity Assessment

#### 2.5.1. Parental Zebrafish Maintenance

Fish maintenance and egg production was carried out as previously described before [23,37].

#### 2.5.2. Zebrafish Embryo Toxicity (ZET) Assay

All experiments were executed in agreement with the guidelines on the protection of experimental animals by the European Council, following the Directive 86/609/EEC, which allows zebrafish embryos to be used up to the moment of free-living. Additionally, our study follows the principles of the Declaration of Helsinki. As so, ZET tests were carried out up to a time post-fertilization (tpf) of 80 h (i.e., within the regulatory limit of exposure, established at 120 h); therefore, no license was required.

After the rinsing and selection process of zebrafish viable zygotes, two hpf -eggs were randomly dispensed into 24-well plates (10 embryos per well at 16-cell stadium, i.e., cleavage period, four replicates per concentration) containing 2 mL/well of incubation medium. The test solutions were renewed every day up to tpf = 80 h. Throughout the ZET experiment, the microplates were kept at 28 °C under a 14:10 light:dark photoperiod cycle. Microplate wells sanitation (i.e., dead embryos removal) was ensured to avoid cross-contamination. ZET experiments were classified as valid when the mortality percentage was inferior to 25%, in the control group (i.e., freshwater as incubation medium).

The developmental age of the zebrafish embryos was measured according to the hpf, and the stage was measured according to Kimmel et al. [31]. Data collection implied microscopic observations and photographic recording at four different time points: 8 h, 32 h, 56 h and 80 h. These time points correspond to crucial developmental stages. In order to avoid bias, random observations were carried out throughout the replicates. All measurements were performed using UTHCSA Image Tool v1.49. Depending on the time post-fertilization, the parameters in the analysis varied. Abnormalities such as deformed body shape, yolk, eyes, heart, atypical cellular masses or atypical pigmentation, and hatching delays were further recorded. All tested concentrations were prepared by dilution in HEPES E3 buffer.

### 2.6. Zebrafish Embryos Microscopy Imaging

#### 2.6.1. Sample Preparation

Four hours and 80 h zebrafish embryos and larvae exposed to 1 mg mL^−1^ fruit-based CD during 2 h, were anesthetized with Tricaine 0.04% prior preservation following a sequenced protocol of fixation (with PFA), permeabilization (with MeOH), rehydration (with milliQ water), re-fixation, glycerol impregnation and analysis in 8-well glass bottom μ-slides (Ibidi, Planegg, Germany).

#### 2.6.2. Fluorescence Microscopy Imaging

The fluorescence microscopy analyses were performed using a wide-field upright fluorescence microscope Nikon Eclipse Ni-E equipped with a Lumencor Sola lamp and ORCA-R^2^ Hamamatsu camera. Images were registered using a 2 × objective and fluorescence filters of 387/11 nm excitation and 447/60 nm emission wavelengths, respectively, and an exposure time of 500 ms.

### 2.7. Statistical Analysis

Statistics were performed using STATISTIC software (StatSoft v.8, Tulsa, OK, USA). Prior to the parametric tests, all data were evaluated for homogeneity of variances using Levene’s test and for normal distribution using the Shapiro-Wilk test. In cases of non-homogeneity, data were transformed before the parametric analysis.

One-way ANOVA was used to analyze the effects of fruit-based CD on zebrafish embryos epiboly (8 hpf), head trunk index (32 hpf), spontaneous movements (32 hpf), hatching (56 hpf), yolk volume (56 hpf) and free-swimming (80 hpf). Nested ANOVA was applied to investigate into the differences of zebrafish embryonic heart rate. To avoid influences associated with covariates, an ANCOVA test was performed to determinate the impact of the nanomaterials on zebrafish embryos yolk volume at tpf = 8 h and 32 h (egg volume was used as co-variable) and on pupil size at 32 hpf (eye size was used as co-variable). At 56 hpf, zebrafish embryos yolk extension (embryo length was used as co-variable) was also analyzed using this statistical approach.

One-way ANOVA model was used to analyze the effect of fruit-based CD on both cell lines tested. Post-hoc comparisons were conducted using Student-Newman-Keuls (SNK). The 0.05 level of probability was considered as the criterion of significance. The graphical data from in vitro tests were generated in GraphPad Prism 6.01.

Please check the Supporting Information for more details.

## 3. Results and Discussion

### 3.1. Characterization of the Fruit-Based Carbon Dots

#### 3.1.1. UV-Vis Absorption and Emission Spectral Characterization of CD

**Figure 1:**
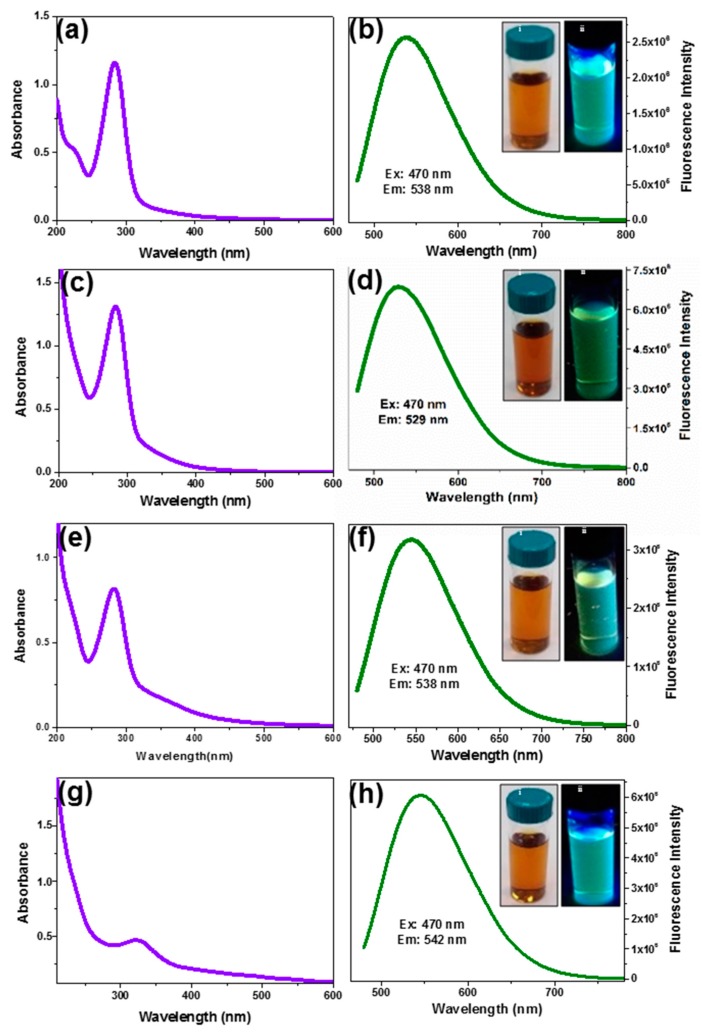
UV-vis and emission spectra of (**a**,**b**) Pear CD, (**c**,**d**) Avocado CD, (**e**,**f**) Kiwi CD and (**g**,**h**) Citrate CD. Inset: Photographs of the corresponding CD under (i) daylight and (ii) UV light.

We used kiwi, avocado and pear as carbon source for the synthesis of fluorescent CD by a facile and ecofriendly hydrothermal method. In order to compare the properties and toxicity of the fruit-based CDs with previously reported materials, we prepared citrate and black pepper CDs using a similar process. The obtained CDs were characterized by UV-vis and fluorescence spectral measurements. Kiwi CDs reveal the absorption peak at 284 nm, avocado CDs at 285 nm, pear CD at 284 nm and citrate CD at 286 nm (Figure 1). The observed absorption bands are attributed to the π-π* electron transition of C=C bonds (sp2 domains) [38,39,40,41]. Black pepper CDs synthesis and their characterizations were reported in our previous publication [36].

Pear, avocado, kiwi, and citrate CDs show a fluorescence emission maxima at 538 (Figure 1b), 529 (Figure 1d), 538 (Figure 1f) and 542 nm (Figure 1h), respectively, with an excitation wavelength of 470 nm. All CDs showed a brownish-yellow color in daylight and green fluorescence under UV-light (insets of Figure 1). All the obtained CDs are stable for more than 6 months in aqueous solution, without any loss of their physicochemical properties, when stored in the dark at 4 °C.

**Figure 2:**
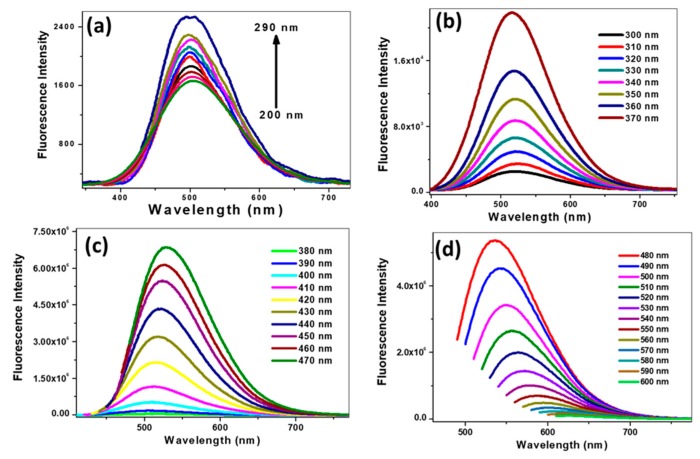
Emission spectra of Avocado CDs with different excitation wavelengths: (**a**) from 200 to 290 nm, (**b**) from 300 to 370 nm, (**c**) from 380 to 470 nm, and (**d**) from 480 to 600 nm (λem: 529 nm; λex: 470 nm).

Figure 2 shows the excitation wavelength-dependent fluorescence emission spectra of avocado CDs. The fluorescence emission spectra of avocado CDs showed a progressive red shift and a dramatic increase of emission intensity when excited with from 200 to 470 nm wavelengths (Figure 2a–c). Beyond 470 nm and up to 600 nm excitation wavelengths, a further red shift was obtained in the emission maximum with a progressive decrease in the emission intensity (Figure 2d). The strongest emission intensity was observed at 529 nm using an excitation wavelength of 470 nm. Hence, we kept 470 nm as the excitation wavelength for further studies with avocado CDs. The emission profile of other synthesized CDs was also studied, and all of them exhibited a similar trend (see Figures S1–S3, Supporting Information). The obtained luminescence of CDs may be attributed to defect states (surface defect emission) and intrinsic defects (zig-zag site emission) [42].

#### 3.1.2. TEM, ζ-Potential, XRD and Raman Spectra of CD

**Figure 3:**
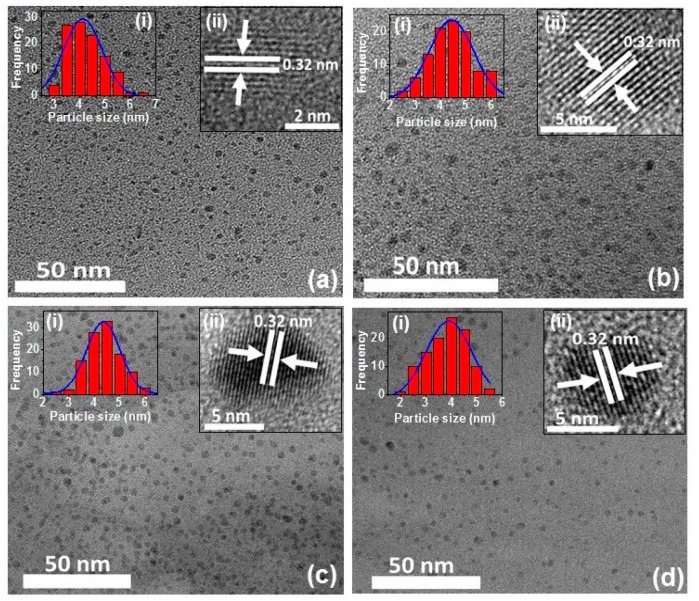
TEM images of (**a**) pear CD, (**b**) avocado CD, (**c**) kiwi CD and (**d**) citrate CD. Insets: (i) Particle size histogram and (ii) the crystalline lattices are identified in each corresponding CD.

Figure 3 shows representative TEM images of the synthesized CDs, demonstrating that they are monodisperse and uniform in size and shape. The average diameters of the CDs were estimated to be 4.12 ± 0.03, 4.42 ± 0.05, 4.35 ± 0.04, and 3.98 ± 0.07 nm for pear, avocado, kiwi, and citrate CD, respectively. The crystal lattices observed by HR-TEM were calculated to display a lattice distance of 0.32 nm (inset of Figure 3), which perfectly matches the previous reports and confirms that the obtained CDs are of crystalline graphitic nature [41,43,44].

The surface charge critically influences the interaction of a nanoparticle with its environment [45]. The synthesized CDs contain -COOH, -OH and epoxides in their structure. These functional groups generate an electrostatic repulsion among CDs [40]. This is the reason why our CDs are stable for several months without agglomeration. All fruit-based c-dots show negative surface charges, which confirms the existence of hydroxyl and carboxylate groups at their surface. Fruit-based CDs’ zeta potential were (ζ mean ± SD)/mV): −14.950 ± 2.871, −8.925 ± 2.167 and −10.100 ± 1.197 for kiwi, avocado and pear CDs, respectively.

Figure S4 (Supporting Information) exhibits the XRD pattern of pear, avocado, kiwi, and citrate CD. The diffraction peaks are observed at 9.5°, 10.1°, 10.2°, and 9.9° respectively, corresponding to the graphitic carbon (001) plane. A broad band was observed around 20°, which corresponds to the graphitic carbon (002) plane [41]. These XRD peaks have a good matching with the characteristic peaks of graphene oxide [41,46,47,48], and are in good agreement with the HR-TEM lattice distances (inset of Figure 3).

**Figure 4:**
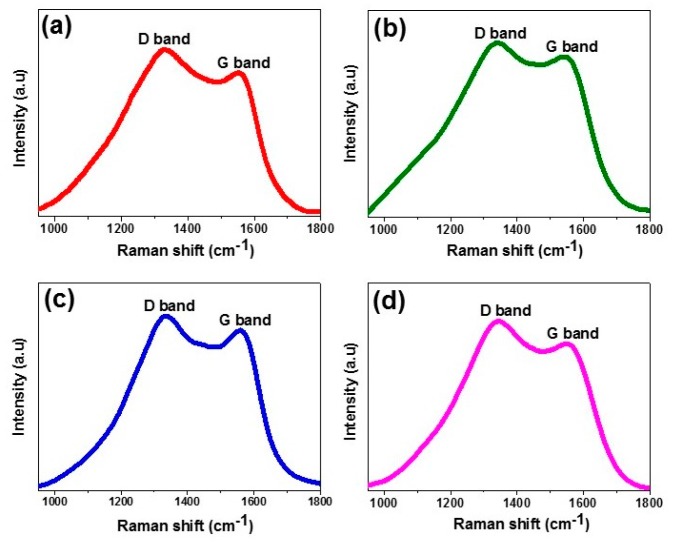
Raman spectra of (**a**) pear CD, (**b**) avocado CD, (**c**) kiwi CD and (**d**) citrate CD.

Raman spectra of the synthesized CD are shown in Figure 4. Pear CDs present a D band at 1331.93 cm^−1^ and a G band at 1555.9 cm^−1^ (Figure 4a). Avocado CDs show the D band at 1341.98 cm^−1^ and the G band at 1547.94 cm^−1^ (Figure 4b). Kiwi CDs show the D band at 1334.48 cm^−1^ and the G band at 1560.0 cm^−1^ (Figure 4c), and finally, citrate CD shows the D band at 1342.71 cm^−1^ and the G band at 1552.88 cm^−1^ (Figure 4d). The obtained D band (sp3) corresponds to the A1g symmetry photons near the K-zone boundary, and the G band (sp2) corresponds to the E2g vibrational mode of sp2 carbon [35,49,50,51]. The relative intensities of D and G bands (ID/IG) for pear, avocado, kiwi, and citrate CDs were 1.15, 1.09, 1.08, and 1.16, respectively, and reveal the existence of vacant lattice sites of sp3 carbon [35,49,50].

#### 3.1.3. Quantum Yield Measurements

The fluorescent quantum yield of each type of synthesized CD was calculated by using the William’s comparative method [51]. For this purpose, quinine sulfate was employed as a reference and the quantum yield was calculated according to Equation (1). *F*_s_ is the integrated fluorescence emission of the sample, *F*_r_ is the integrated fluorescence emission of the reference, *A*_r_ is the absorbance at the excitation wavelength of the reference, *A*_s_ is the absorbance at the excitation wavelength of the sample, *QY*_s_ is the quantum yield of the sample, and *QY*_r_ is the quantum yield of the reference fluorophore (quinine sulfate *QY* = 54%). The calculated fluorescence quantum yields of pear, avocado, kiwi, and citrate CD are 20, 35, 23, and 35%, respectively. The obtained high quantum yield values confirm that the synthesized CDs are highly fluorescent. Avocado and citrate CD showed the highest quantum yield among all other synthesized CDs. A summary of the characteristic parameters studied for each CD is collected in Table 1.

(1) $$QY_{s}=\frac{F_{s}A_{r}QY_{r}}{F_{r}A_{s}}$$

### 3.2. In Vitro Cytotoxicity of Fruit-Based Carbon Dots

One of the most important parameters to evaluate the applicability of the fruit-based CD is the toxicity level induced by these nanomaterials when interacting with cells. Therefore, the cytotoxic effect of the fruit-based CD was evaluated. Also, the potential anticancer activity of novel fruit-based carbon dots was investigated by studying their differential activity against normal epithelial cells (HK-2) and colorectal cancer (Caco-2) cells. The cytotoxicity of the nanomaterials was evaluated using PrestoBlue^®^ cell viability reagent (PB) after exposure for 48 and 72 h to growing concentrations of the CD. In all cases, the highest concentration of HEPES E3 buffer (medium control) was 45% and it did not produce any significant effect on cellular viability, for either of the tested cell lines (data not shown). PB fluorescence was directly proportional to cell density, thus it was used to calculate the percentage of cell viability, assuming 100% as the viability obtained for the vehicle control.

The results obtained were compared with CD synthesized from citric acid as a commercial source control and black pepper as a non-fruit control with previously reported potential anticancer activity and bioimaging application.

**Figure 5:**
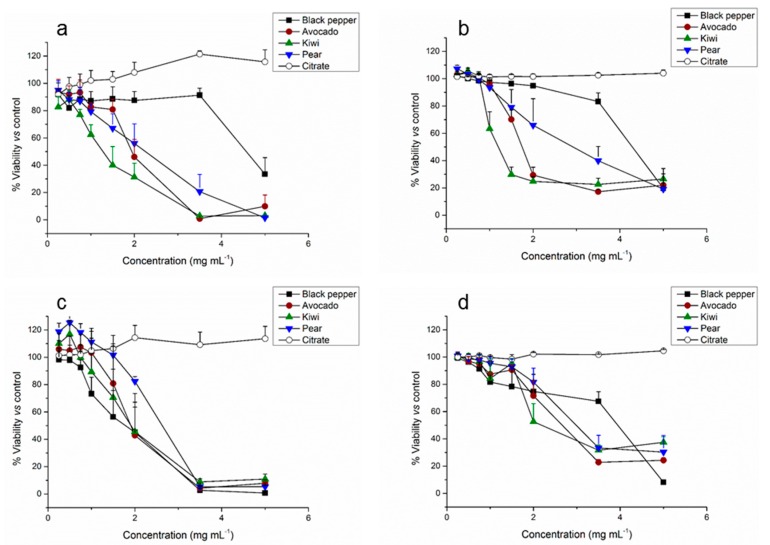
Cell viability evaluation after 48 h (**a**,**c**) and 72 h (**b**,**d**) incubation with growing concentrations of each CD. Results are expressed as viability mean ± SEM of four and seven independent experiments for Caco-2 (**c**,**d**) and HK-2 (**a**,**b**) respectively.

Figure 5 shows the dose-dependent in vitro cytotoxicity of CD for the tested cell lines at 48 and 72 h. The fruit-based CD-induced obvious cytotoxicity to the Caco-2 cell line when their concentration was higher than 1.5 mg mL^−1^ can be noted. These results are in agreement with a previous work using mango-based CD, where A-549 (human lung carcinoma) cells showed nearly 100% cell viability up to 2 mg mL^−1^ [52]. Also, HeLa (human cervix epithelial) cells viability remained unchanged when the concentration of glycerol-based CD increased from (0 to 1.14) mg mL^−1^ [53]. When compared to the citric acid-derived (citrate) CD used as a commercial reference (synthesized as described in previous work [54]), they did not induce any effect, as expected. Unlike the other CDs prepared in this study, citrate CDs were not prepared from a food matrix but rather citric acid. Thus, only the products of citric acid decomposition could be present in the CD. We consider these CDs to be representative controls due to the fact that the source would not influence much of the biological activity, as already reported in our previous work, where no toxicity was observed in any of the cell lines tested in vitro [36]. However, pepper CD [24] (used as non-fruit reference) were more toxic for cancer cells, where concentrations above 0.5 mg mL^−1^ lead to more than 50% mortality, which is in agreement with previous studies using other spicy-based CDs [7,35] and a recent study that reported this effect [24].

When testing the same concentrations of the fruit-based CD in HK-2 cells, in vitro cytotoxicity analyses demonstrated the stronger negative effect of the fruit-based CD on normal cells proliferation, although, in general, no more than 25% of mortality was observed for concentrations up to 1 mg mL^−1^. Sun’s group also demonstrated that bare CD were not toxic to normal cells up to a relatively high concentration of 0.4 mg mL^−1^ [9]. It also became evident that the effects of pepper CD in HK-2 cells were clearly less pronounced than the ones observed for Caco-2 cells. The fact that citrate CD did not induce any significant effect on cell viability, neither on Caco-2 nor HK-2 cells, together with the different toxic profile obtained with pepper CD versus fruit-based CD suggests that the inhibition effect on cellular growth can be attributed to the different sources employed for the CD synthesis. In fact, very recently, Pierrat et al. claimed that the toxicity of the CD is mainly determined by the synthesis source [55,56].

The results in Figure 5 show that, globally, the toxicological profile obtained at 48 h exposure was maintained after 72 h suggesting that there was no progression on the cellular toxicity and that the toxic effect of the fruit-based CD was accomplished during the first 48 h.

In general, pear CD showed to be the less toxic, while kiwi CD demonstrated to be the most toxic. It is clear, that overall, the toxicity of the fruit-based CD in cancer cells was lower than in normal cells, as it can be observed comparing the lethal dose 50 (LD50) values obtained (Table 2).

The unique proprieties exhibited by CD are known to result in a variety of interactions with cells, leading to potential necrosis, apoptosis, inflammation, oxidative stress, and other toxic responses [56,57,58,59]. However, in the present work, the mechanisms ruling the cytotoxic effect exerted by these fruit-based CDs were not studied and should be further explored.

### 3.3. In Vivo Toxicity of Fruit-Based Carbon Dots

**Figure 6:**
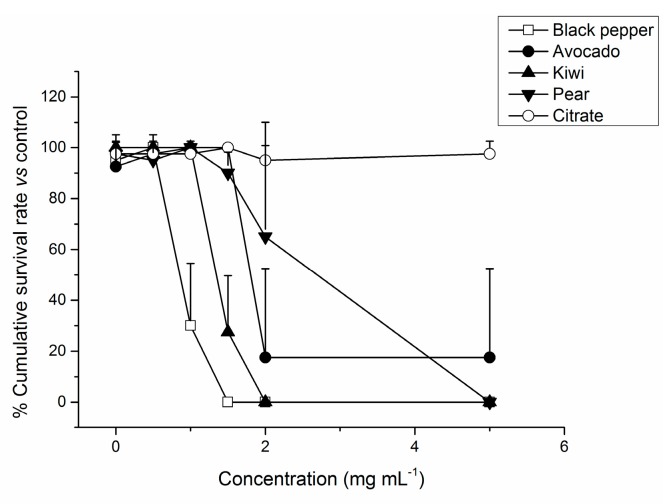
The cumulative survival rate of zebrafish embryos at 80 h when exposed to different concentrations of kiwi CD. Results represent the CSR mean ± SD. Statistical significance represented for *t*_pf_ = 80 h.

Zebrafish eggs of two hours were exposed to different concentrations of the fruit-based CD over 3 days (Figure 6). The results obtained with fruit-based CD were also compared with two other CDs synthesized from sources other than fruit sources: citric acid as a reference previously reported as innocuous, and black pepper. Black pepper CDs were recently studied in vitro and postulated as anticancer materials [36].

When analyzing the lethal effect of the fruit-based CD, a dose-dependent mortality (check Table S2 for statistical significance) only from 1.5 mg mL^−1^ onwards was verified. In agreement with the data obtained with in vitro experiments, kiwi CD showed higher embryotoxicity than the other fruit-based CDs, while citrate CD did not cause any apparent toxicity. For the pepper CD, a clear embryonic lethality was obtained above 0.5 mg mL^−1^.

Then, the sub-lethal toxicity of the CD was studied. Concentrations that induced more than 25% mortality were excluded from the sub-lethal toxicity study [60].

Table 3 summarizes the effect on the different parameters monitored during the zebrafish embryo’s development (8 h to 80 h), giving an overall and important perspective of the fruit-based CD’ potential sub-lethal toxicity. At the initial stages of zebrafish embryogenesis, none of the fruit-based CDs seemed to cause any delay in development. Even so, further effects on spontaneous movements, free swimming and heart rate were identified, suggesting that these nanomaterials have the potential to disrupt features of the zebrafish early life neuro-motor coordination [61]. Moreover, the results obtained with pear and avocado CD point to an inhibition of zebrafish embryos’ nutrient absorption from their yolk sacs. Due to the nutrients present in their yolks, zebrafish embryos do not need food for up to 7 days. As a consequence of using this nutritional reserve, their yolk volume tends to decrease over the embryonic development [29,62]. The yolks of zebrafish embryos exposed to pear and avocado CD from 1 mg mL^−1^ and higher were statistically different (i.e., larger) from the control group, implying that the embryos were not getting the required nutrients [24] (pear: One-way ANOVA: F (3,76) = 18.626; *p* < 0.05; avocado: One-way ANOVA: F (5,109) = 3.889; *p* < 0.05). Also, a delay in the hatching rate was observed, which may be a sign of disruption in chorionase activity [63]. In line with the in vitro results, zebrafish embryos exposed to citrate CD did not show morphological malformations, not even for 7 days at the higher concentration tested. Pepper CD were found to be non-toxic up to 0.5 mg mL^−1^ since no retardation and development defects were detected at this concentration, but highly toxic at higher concentrations. Interestingly, in vitro we obtained similar results to the work of Reference [36]; black pepper CDs exhibited higher toxicity against cancer cells than normal cells and were almost innocuous to normal cells for concentrations of up to 3 mg mL^−1^. This activity pointed to a potential anticancer activity of black pepper CDs that could be related to the presence of piperine (or its decomposition products), an alkaloid with antioxidant properties that was recently reported as potential anticancer agent in in vitro studies. We demonstrated the presence of a trace amount of piperine in the black pepper CDs solution in our previous work. Some studies showed that piperine induces higher toxicity in vivo than in vitro due to its inhibitory effect on P450 cytochrome, which is responsible for the metabolism of many drugs [64]. Additionally, piperine was shown to disturb lipids’ metabolism, which is crucial in the zebrafish’s (and all vertebrates) embryo development [65]. Taking into account only the in vitro results with cells, black pepper CDs could be considered to be used up to 3 mg mL^−1^ for bioimaging or therapeutics. However, our in vivo results allowed us to re-evaluate the Non-Observed Adverse Effect Level (NOAEL) for those CDs.

The diverse fruit-based CD showed slightly different in vivo toxicity, with kiwi CD being the most toxic (LD50 = 1.44 mg mL^−1^) and pear CD the last toxic (LD50 = 2.22 mg mL^−1^) (Table 2). The fact that citrate CD did not induce any significant effect on zebrafish embryo development and the different profiles obtained with pepper and fruit-based CD reinforce the idea that depending on the starting material employed for CD synthesis, different toxic responses are obtained.

### 3.4. Imaging of Zebrafish Embryos Incubated with Fruit-Based Carbon Dots

Zebrafish embryos with tpf equal to 4 and 80 h exposed to 1 mg mL^−1^ of fruit-based CD for 2 h were used as a model to validate the in vivo imaging application of the fruit-based CD. Considering the toxicity results obtained in vivo, 1 mg mL^−1^ was defined as a suitable concentration. No significant fluorescent signal was observed in 4 h exposed zebrafish embryos, although a slighter fluorescence was observed in the zebrafish embryos incubated with avocado CD, mainly around the chorion but inside as well (data not shown). This may be indicative of the fact that fruit-based CDs were retained in this structure or only partially internalized into the embryos, at least in the first 2 h of exposure. These results could suggest low retention of the fruit-based CDs in the zebrafish embryos or provide an indication of the aggregation of the fruit-based CD, which would make their internalization difficult [66].

**Figure 7:**
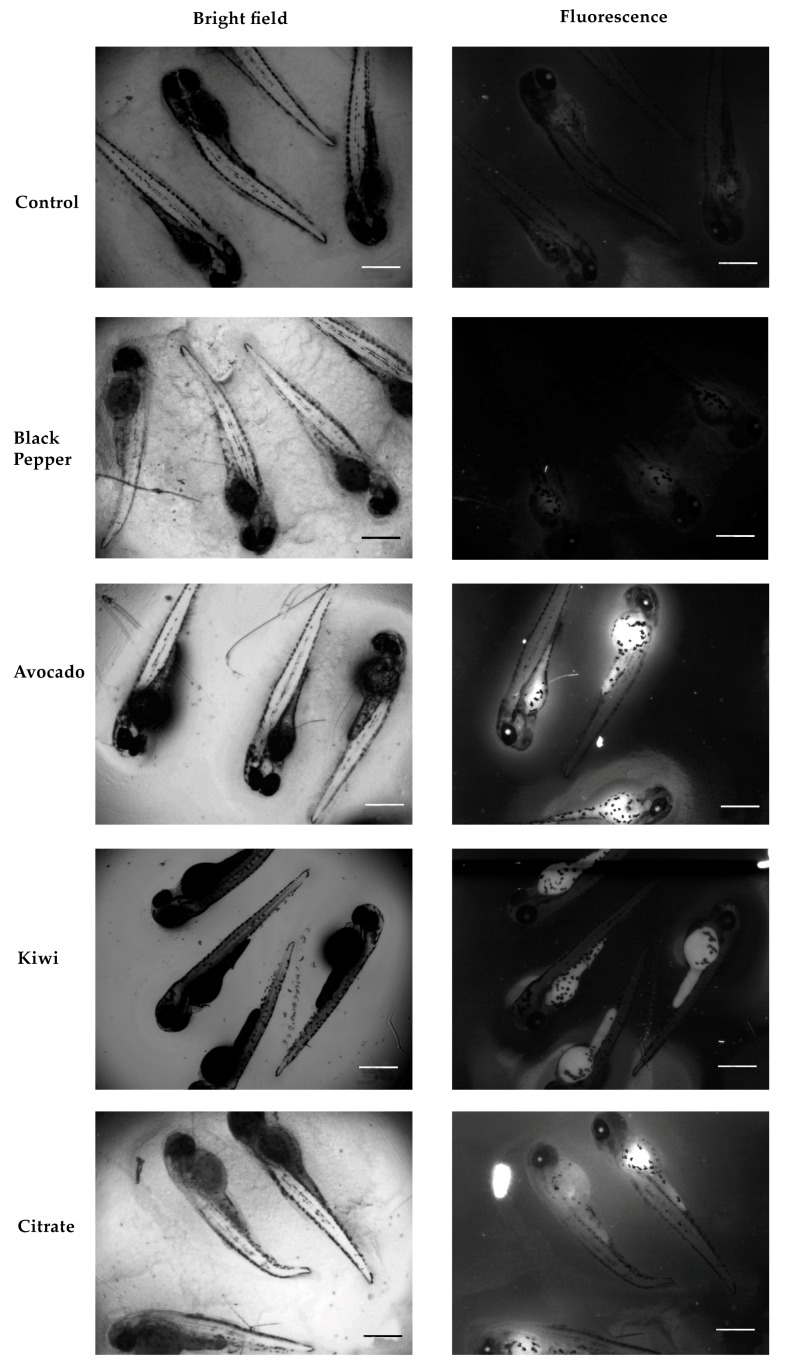
Bright field (left panel) and fluorescence images (right panel) of 80 h old zebrafish embryos after incubation for 2 h with 1 mg mL^−1^ except for black pepper, which concentration was 0.5 mg mL^−1^. Scale bars = 500 μm.

In Figure 7, a more intense fluorescence was observed in the zebrafish embryos incubated with avocado and citrate CD. Huang et al. demonstrated similar fluorescent images collected at a similar excitation wavelength (555 nm) using zebrafish larvae incubated with 1.14 mg mL^−1^ of glycerol-based CD in real time [40]. Our data further demonstrates that yolk and eyes are indeed hot-spots for fruit-based CD bioaccumulation. It has already been shown that CD seemed to enter into the zebrafish larvae body through skin adsorption [52,66] and accumulate especially in the yolk sac, yolk extension and eye. This reveals their high affinity for lipids, which, together with their fluorescent properties, could be useful to elucidate different aspects of lipoprotein and nutritional biology in lipid transport and metabolism [67]. Overall, the different fluorescence observed is in agreement with the quantum yield (i.e., efficiency of fluorescence) values of the CD tested [40]. Avocado CD presented the higher quantum yield (35%), followed by citrate CD (35%) and kiwi CD the lowest (23%), using quinine sulfate as the reference standard. Pear CD were not analyzed for their bioimaging proprieties because of their low quantum yield value compared with the others (20%). The fruit-based CD fluorescence intensity was clearly higher for zebrafish embryos at 80 h than those at 4 h, which could indicate a more efficient uptake by the more developed embryos. On the other hand, the contribution of chorion as a barrier to the entrance of the CD changes along development. Therefore, it would be interesting to further investigate the bioimaging of 4 h zebrafish embryos without chorion (i.e., dechorionated) exposed to the fruit-based CD. Fluorescence microscopy represents a highly useful tool to investigate the chorion in its function as a potential barrier to the uptake of chemicals [68].

Despite many existing studies in the literature reporting on CD synthesized from natural sources and used in bioimaging, very few of them have been demonstrated as good candidates for in vivo imaging and even, more limited number of studies have reported their in vivo toxicity (Table S2).

## 4. Conclusions

In the present study, three different CDs were synthesized from kiwi, pear and avocado fruits, by one green-pot hydrothermal method obtaining materials with a relatively high fluorescent yield. Fruit-based CD from pear, avocado and kiwi showed slightly lower toxicity in human epithelial cancer cells than in normal cells. Opposite results were obtained with black pepper CD (other food-based CD), and showed potential as anti-cancer materials. In vitro data showed that only high doses of fruit-based CD, i.e., above 1.5 mg mL^−1^, induced noteworthy cell death, suggesting their biocompatibility for lower concentrations. Also, when monitoring the early life of zebrafish in vivo, no sub-lethal signs of toxicity were detected for concentrations up to 1.5 mg mL^−1^, demonstrating the low toxicity of fruit-based CD in comparison with metal q-dots reported in the literature [13]. In this way, CD turns out to be an equivalent, low-cost and eco-friendly substitute for metal q-dots.

Differential toxicity of CD from different food sources was demonstrated and should be further explored, as it could be dependent on the food matrix used for CD synthesis. Despite the fact that kiwi, avocado and pear CDs did not present any significant anti-cancer activity in this report, we observed significant differences in their toxicity. Further studies are envisioned in order to unravel the possible mechanisms of their observed biological activity.

Furthermore, avocado CD demonstrated high potential for in vivo fluorescence bioimaging, as shown in zebrafish embryos with *t*_pf_ = 80 h. Without any modification in their surface for tissue specificity, avocado CD are internalized and retained especially in the eyes and yolk sac, thus being potentially useful as a fluorescent contrast agent and/or lipid metabolism fluorescent probe.

Finally, very few studies in the literature have investigated the toxicity profile of newly synthesized CDs in vitro and in vivo before their application for bioimaging. In our study, the importance of this step is stressed by the results obtained with the black pepper CDs, which despite showing low toxicity at mg mL^−1^ concentrations in vitro, showed great toxicity in vivo in that range. Diverse studies report cytotoxicity of CDs and move forward directly to the in vivo imaging studies using mice and applying concentrations of CDs that might be unsafe for the animal.

## Figures and Tables

**Table 1:** Summary of parameters measured for characterization of the synthesized CD.

Parameters	Pear CD	Avocado CD	Kiwi CD	Citrate CD
*λ*_ex//_*λ*_em_ (nm)	470/538	470/529	470/544	470/546
FWHM (nm)	105	109	111	117
Stokes shift (nm)	68	59	74	76
Under UV light	Green emission	Green emission	Green emission	Green emission
Raman (I_D_/I_G_)	1.15	1.09	1.08	1.16
TEM size (nm)	4.12 ± 0.03	4.42 ± 0.05	4.35 ± 0.04	3.98 ± 0.07
Quantum Yield (%)	20	35	23	35

**Table 2:** Lethal dose i.e., *LD_50_* of kiwi CD, pear CD, avocado CD, and pepper CD after 72 h exposure to Caco-2 cells and HK-2 cells and after 80 h exposure to zebrafish embryos.

	Kiwi	Pear	Avocado	Black Pepper
In vitro (Caco-2)	2.246 ± 0.043 *R*^2^ = 0.742	3.276 ± 0.030 *R*^2^ = 0.804	2.680 ± 0.030 *R*^2^ = 0.858	2.326 ± 0.042 *R*^2^ = 0.757
In vitro (HK-2)	1.340 ± 0.115 *R*^2^ = 0.725	2.072 ± 0.246 *R*^2^ = 0.745	1.839 ± 0.108 *R*^2^ = 0.891	4.054 ± 0.071 *R*^2^ = 0.979
In vivo	1.444 ± 2.756 *R*^2^ = 0.975	2.224 ± 0.074 *R*^2^ = 0.996	1.964 ± 0.131 *R*^2^ = 0.962	0.985 ± 0.119 *R*^2^ = 0.998

**Table 3:** Zebrafish embryotoxicity testing. (+) corresponds to a statistically significant effect and (−) corresponds to a non-significant effect on tested variables.

	*t*_pf_/h	Independent Variables	Kiwi 1 mg mL^−1^	Pear 1.5 mg mL^−1^	Avocado 1 and 1.5 mg mL^−1^	Citrate 5 mg mL^−1^	Black Pepper 0.5 mg mL^−1^
Morphometric analysis	8	Epibolic arc	−	−	−	−	−
	8–56	Yolk volume	−	+	+	−	−
	32	Head-trunk angle	−	−	−	−	−
	56	Eye surface	−	+	−	−	−
	56	Hatching	−	+	+	−	−
Neuro-motor coordination	32	Cardiac frequency	+	+	+	−	−
	32	Spontaneous movements	+	−	+	−	−
	80	Free-swimming	+	+	+	−	−

## Supplementary Materials

The following are available online at http://www.mdpi.com/2079-4991/9/2/199/s1, Figure S1: Emission spectra of pear CD under different excitation wavelengths, Figure S2: Emission spectra of kiwi CD under different excitation wavelengths, Figure S3: Emission spectra of citrate CD under different excitation wavelengths, Figure S4: XRD pattern of CDs, Figure S5: Caco-2 cell viability evaluation after 48 and 72 h incubation with growing concentrations of pepper CD, Figure S6: Caco-2 cell viability evaluation after 48 h and 72 h incubation with growing concentrations of avocado CD, Figure S7: Caco-2 cell viability evaluation after 48 h and 72 h incubation with growing concentrations of kiwi CD, Figure S8: Caco-2 cell viability evaluation after 48 h and 72 h incubation with growing concentrations of pear CD, Figure S9: Caco-2 cell viability evaluation after 48 h and 72 h incubation with growing concentrations of citrate CD, Figure S10: HK-2 cell viability evaluation after 48 h and 72 h incubation with growing concentrations of pepper CD, Figure S11: HK-2 cell viability evaluation after 48 h and 72 h incubation with growing concentrations of avocado CD, Figure S12: HK-2 cell viability evaluation after 48 h and 72 h incubation with growing concentrations of kiwi CD, Figure S13: HK-2 cell viability evaluation after 48 h and 72 h incubation with growing concentrations of pear CD, Figure S14: HK-2 cell viability evaluation after 48 h and 72 h incubation with growing concentrations of citrate CD, Table S1. Autofluorescence of CD in Caco-2 and HK-2 culture medium. Table S2. Properties of the natural sourced CDs used in bioimaging and reported in the literature. All of them were tested for cell bioimaging. Table S3. Statistical analysis equations for the diverse sub-lethal toxicity parameters studied in zebrafish embryos.
