# Supplementary material for: Biocompatibility and Bioimaging Potential of Fruit-Based Carbon Dots
Source: Nanomaterials (Basel). 2019 Feb 3;9(2):199. doi: 10.3390/nano9020199 (PMC6409625; doi:10.3390/nano9020199)
Supplement: Supplementary file 1 [file nanomaterials-09-00199-s001.pdf]

Supporting Information

Biocompatibility and Bioimaging Potential of Fruit-Based Carbon Dots

Cindy Dias [1,2,†], Vasimalai Nagamalai [1,3,†], Marisa P. Sárria [1,*], Ivone Pinheiro [1], Vânia Vilas-Boas [1,4]. João Peixoto [2] and Begoña Espiña [1,*]

[1] INL - International Iberian Nanotechnology Laboratory, Braga, 4715-330, Portugal; cindydias93@gmail.com (C.D.); vasimalai.gri@gmail.com (V.N.); ivone.pinheiro@inl.int (I.P.); vfevilasboas@gmail.com (V.V.-B.)

[2] CEB – Centre of Biological Engineering, University of Minho, 4720-057 Braga, Portugal; jmp@deb.uminho.pt

[3] Department of Chemistry, B.S. Abdur Rahman Crescent Institute of Science and Technology, Vandalur, Chennai-600048, India

[4] UCIBIO-REQUIMTE, Laboratory of Toxicology, Biological Sciences Department, Faculty of Pharmacy, University of Porto, Rua de Jorge Viterbo Ferreira, 228, 4050–313 Porto, Portugal

† These authors contributed equally to this work.

* Correspondence: marisa.passos@inl.int (M.P.S.); begona.espina@inl.int (B.E.)

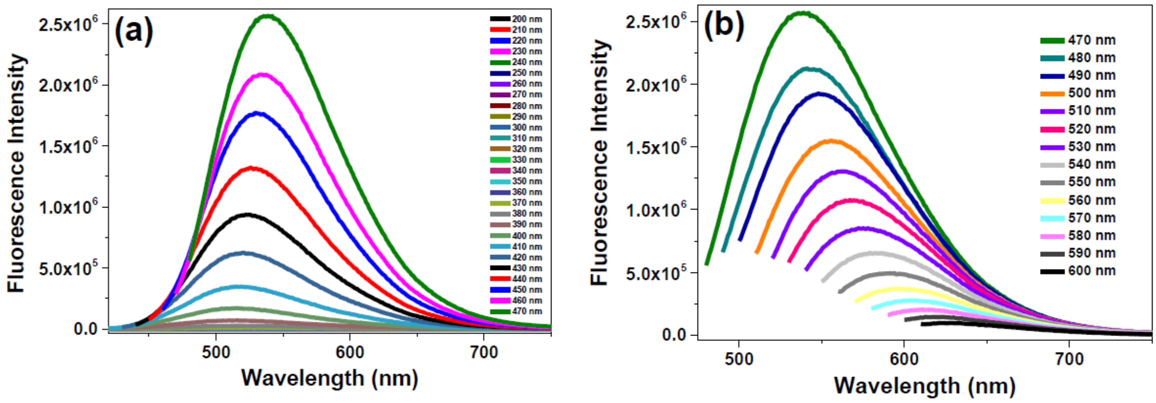

Figure S1: Emission spectra of pear CD under different excitation wavelengths (**a**) from 200 to 470 nm and (**b**) from 470 to 600 nm. Optimum selected conditions are λex/λem: 470/538 nm.

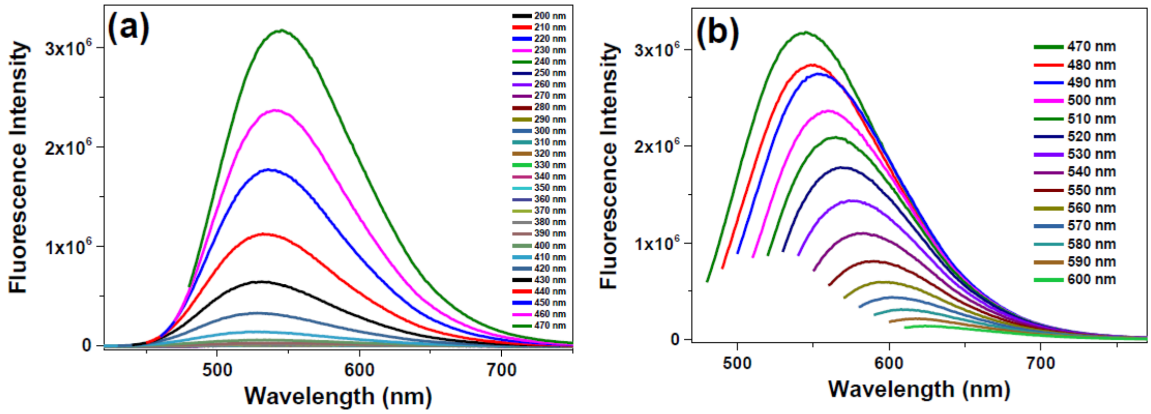

Figure S2: Emission spectra of kiwi CD under different excitation wavelengths (**a**) from 200 to 470 nm and (**b**) from 470 to 600 nm. Optimum selected conditions are λex/λem: 470/544 nm.

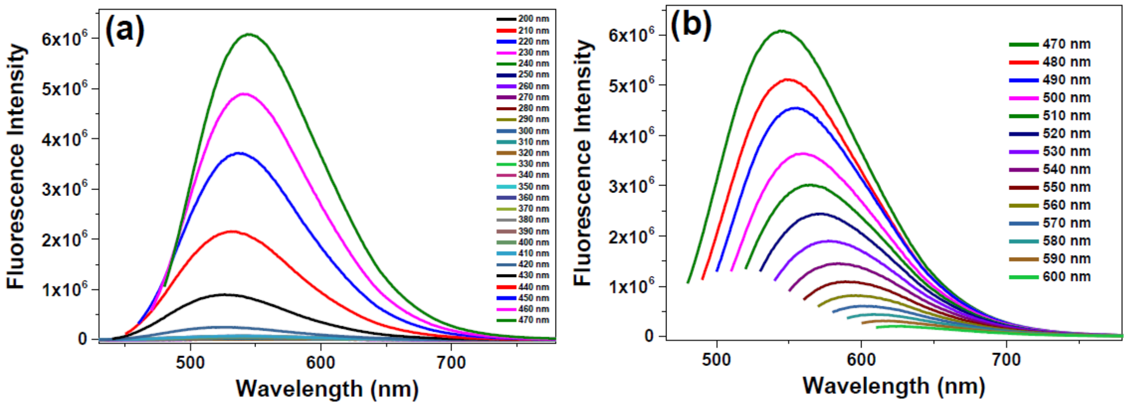

Figure S3: Emission spectra of citrate CD under different excitation wavelengths (**a**) from 200 to 470 nm and (**b**) from 470 to 600 nm. Optimum selected conditions are λex/λem: 470/546 nm.

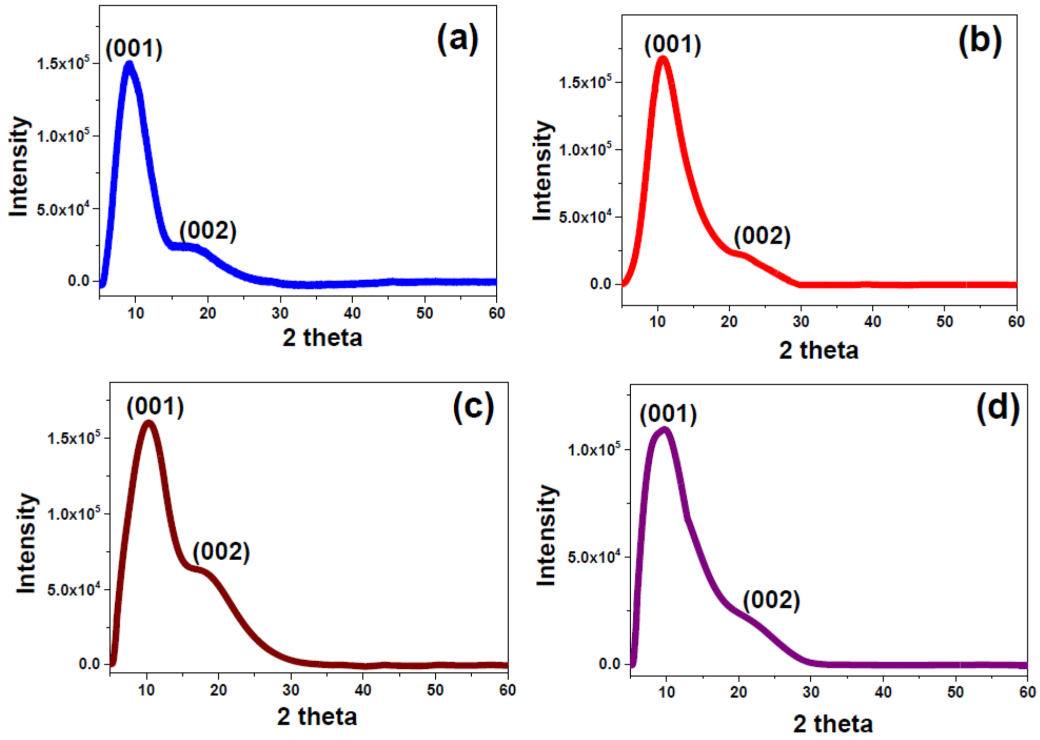

Figure S4. XRD pattern of (**a**) Pear CD, (**b**) Avocado CD, (**c**) Kiwi CD and (**d**) Citrate CD.

Table S1. Autofluorescence of CD in Caco-2 and HK-2 culture medium (A.U.).

Caco-2 cells	*Fluorescence*	*HK-2 cells*	*Fluorescence*
Control	3136.5	*Control*	6142
Kiwi	3802	*Kiwi*	4152
Pear	3111	*Pear*	6550
Avocado	3188	*Avocado*	5104
Citrate	3647	*Citrate*	6952
Pepper	3802	*Pepper*	5519

Table S2. Properties of the natural sourced CDs used in bioimaging and reported in the literature. All of them were tested for cell bioimaging.

**Precursor**	**Synthesis method**	**Time**	**Size**	**QY (%)**	***In vivo* imaging/toxicity**	**Ref**
Apple juice	Hydrothermal	12	4.5	4.27	X / X	1
Bagasse	Hydrothermal	3	1.8	12.3	X / X	2
Bee pollens	Hydrothermal	24	1.1-2.1	6.1-12.8	X / X	3
**Black pepper**	Hydrothermal	12	3.5 ± 0.1	43.6	√ / √	4 and this work
Bombyx mori silk	Hydrothermal	3	5	13.9	X / X	5
Bread	Acid oxidation	4	2-10	4.5	X / X	6
Cabbage	Hydrothermal	5	2-6	16.5	X / X	7
Carica papaya juice	Hydrothermal	12	3	7	X / X	8
Coffee grounds	Heating	2	5±2	3.8	X / X	9
Cow manure	Chemical oxidation	72	4.8	65*	X / X	10
Curcumine	Hydrothermal	12	3.28	8.6	√ / √	11
Dried shrimp	Hydrothermal	12	6	54*	X / X	12
Enteromorpha prolifera	Hydrothermal	3-10	2.75±0.12	8	X / X	13
Garlic	Hydrothermal	3	11	17.5	X / X	14
Garlic	Microwave	2min	5	5	X / X	15
Ginger	Hydrothermal	2	4.3	13.4	X / √	16
Grape juice	Hydrothermal	12	2.7±0.5	13.5	X / X	17
Hair fibre	Acid treatment	24	2-10	11.1	X / X	18
Honey	Hydrothermal	2	2	19.8	X / X	19
Konjac flour	Pyrolysis	1.5	3.37	13/22	X / X	20

Lemon juice	Hydrothermal	10	4.6	28	√ / X	21
Lychee seed	Carbonization	2	1.12	10.6	X / X	22
Mango	Carbonization	0.3-1	5-15	0.48-3.92	√ / √	23
Milk	Hydrothermal	2	3	12	X / X	24
Milk	Hydrothermal	2-8	3-5	5.86	X / X	25
Neem gum	Biogenic	3	5-8		X / X	26
Nescafe	Heating	0.25	4.4	5.5	√ / X	27
Orange juice	Hydrothermal	2.5	1.5-4.5	26	X / X	28
Onion waste	Hydrothermal	2	7-25	28	X / X	29
Onion peel	Microwave	1-3min	2-4		X / X	30
Papaya	Hydrothermal	5	2-6/8-18	18.39-18.98	X / X	31
Peanut shell	Carbonization	2	0.4-2.4	9.91	X / X	32
Pigskin	Hydrothermal	2	3.5-7.0	24.1	X / X	33
Plant soot	Reflux with acid	20	2-4.3	0.72-4.28	√ / X	34
Potato	Hydrothermal	12	0.2-2.2	6.14	X / X	35
Sugar cane juice	Hydrothermal	3	2.71	5.76	X / X	36
Sweet potato	Hydrothermal	18	2.5-5.5	8.64	X / X	37
Trapa bispinosa peel	Thermal oxidation	2	5-10	1.2	X / X	38
Vitamin B1	Carbonization	2	1-6	76*	X / X	39
Waste frying oil	Heating with acid	5min	1-4	3.66	X / X	40
Watermelon peels	Carbonization	2	2	7.1	X / X	41
**Avocado juice**	Hydrothermal	12	4.42 ± 0.05	35	√ / √	This work
**Kiwi juice**	Hydrothermal	12	4.35 ± 0.04	23	√ / √	This work
**Pear juice**	Hydrothermal	12	4.12 ± 0.03	20	√ / √	This work

Statistical Analysis

Statistics were performed using STATISTIC software (StatSoft v.8, US). Prior to the parametric tests all data were evaluated for homogeneity of variances using Levene's test and for normal distribution using Shapiro-Wilk test. In cases of non-homogeneity, data were transformed before the parametric analysis.

One-way ANOVA was used to analyze the effects of fruit-based CD on zebrafish embryos epiboly (8 h_{pf}), head trunk index (32 h_{pf}), spontaneous movements (32 h_{pf}), hatching (56 h_{pf}), yolk volume (56 h_{pf}) and free-swimming (80 h_{pf}). Nested ANOVA was applied to investigate differences on zebrafish embryonic heart rate. To avoid influences associated with covariates, ANCOVA test was performed to determinate the impact of the nanomaterials on zebrafish embryos yolk volume at t_{pf} = 8 h and 32 h (egg volume was used as co-variable) and on pupil size at 32 h_{pf} (eye size was used as co-variable). At 56 h_{pf}, zebrafish embryos yolk extension (embryo length was used as co-variable) was also analyzed using this statistical approach.

One-way ANOVA model was used to analyze the effect of fruit-based CD on both cell lines tested. Post-hoc comparisons were conducted using Student-Newman-Keuls (SNK). The

0.05 level of probability was considered as criterion of significance. The graphical data from *in vitro* tests were generated in GraphPad Prism 6.01.

Table S3. Statistical analysis equations for the diverse sub-lethal toxicity parameters studied in zebrafish embryos.

	hpf	Independent variables	Statistical test	Kiwi	Pear	Avocado	Citrate	Pepper
**Morphometric analysis**	8	Epibolic arc	One-way ANOVA	*F* (5,110)=1.881 *P*= 0.103	*F* (3,73)= 0.804; *P*=0.496	*F* (5,112)= 0.713; *P*=0.615	*F* (5,107)=2.999; *P*<0.05	*F* (1, 38)=0.422, *P*=0.520
	8-56	Yolk volume	ANCOVA	*F* (5,72)= 2.985; *P*<0.05	*F*=(3,72)=1.669; *P*= 0.248	*F* (5,111)=10.741; *P*<0.05	*F* (5,106)=0.853; *P*=0.516	*F* (1,36)=1.696; *P*=0.201
	32	Head-trunk angle	One-way ANOVA	*F* (5,72)= 2.791; *P*<0.05	*F* (3,54)= 2.099; *P*=0.111	*F* (5,81)= 2.966; *P*<0.05	*F* (5,75)= 1.969; *P*=0.093	*F* (1,27)= 0.358, *P*=0.554
	56	Eye surface	One-way ANOVA	*F* (5,104)=7.389; *P*<0.05	*F* (3,27)=37.105; *P*<0.05	*F* (5,109)=10.228, *P*<0.05	*F* (5,106)=3.227; *P*<0.05	*F* (1,36)=9.000, *P*<0.05
	56	Hatching	One-way ANOVA	*F* (5,14)= 10.962; *P*<0.05	*F* (3,8)= 8.569; *P*<0.05	*F* (5,12)= 5.780; *P*<0.05	*F* (5,12)= 1.749; *P*=1.980	*F* (1,4)= 1.662, *P*=0.267
**Neuro-motor coordination**	32	Cardiac frequency	Nested ANOVA	*F* (7,119)= 65.515; *P*<0.05	*F* (5,83)= 45.328; *P*<0.05	*F* (6,108)= 116.21; *P*<0.05	*F* (6,108)= 281.23; *P*<0.05	*F* (2,36)= 49.768, *P*<0.05
	32	Spontaneous movements	One-way ANOVA	*F* (5,14)= 3.749; *P*<0.05	*F* (3,8)= 1.8678; *P*=0.213	*F* (5,12)= 5.735; *P*<0.05	*F* (5,12)= 5.049; *P*<0.05	*F* (1,4)= 1.077, *P*=0.358

	80	Free-swimming	One-way ANOVA	F (6,14)= 81.584; P<0.05	F(3,8)= 32.000; P<0.05	F (5,12)= 113.80; P<0.05	F (5,12)= 10.677; P<0.05	F (1,4)= 27.000;P<0.05
	80	Survival	Chi-square	χ^2=6.848; DF=6; P=0.335	χ^2 = 100.294, DF=5; P<0.05	χ^2= 125.864, DF=7, P<0.05	χ^2 =0.128; DF=5; P=0.999	χ^2= 306.333; DF = 5; P<0.05

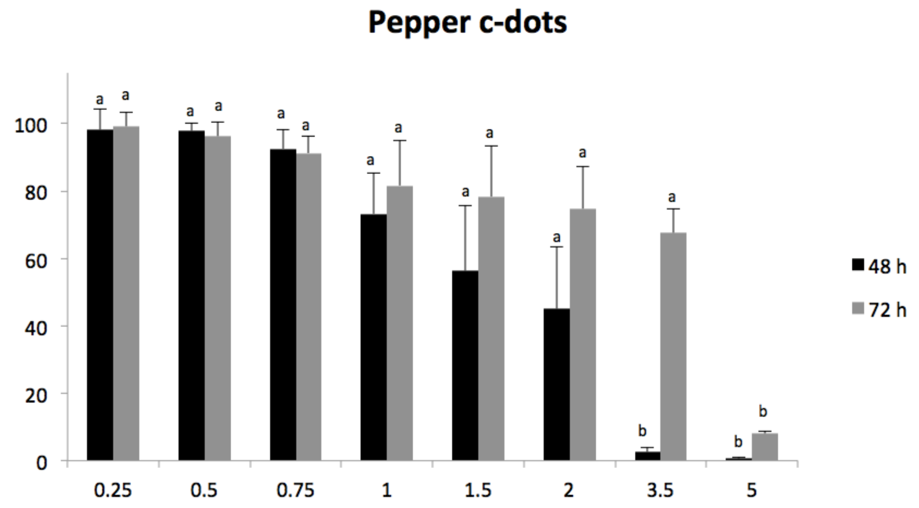

Figure S5. Caco-2 cell viability evaluation after 48 and 72 h incubation with growing concentrations of pepper CD. Different letters indicate significant differences among treatments (P<0.05). 48 h: F(8, 26)=14.885, P<0.05. 72 h: F(8, 27)=8.4291, P<0.05.

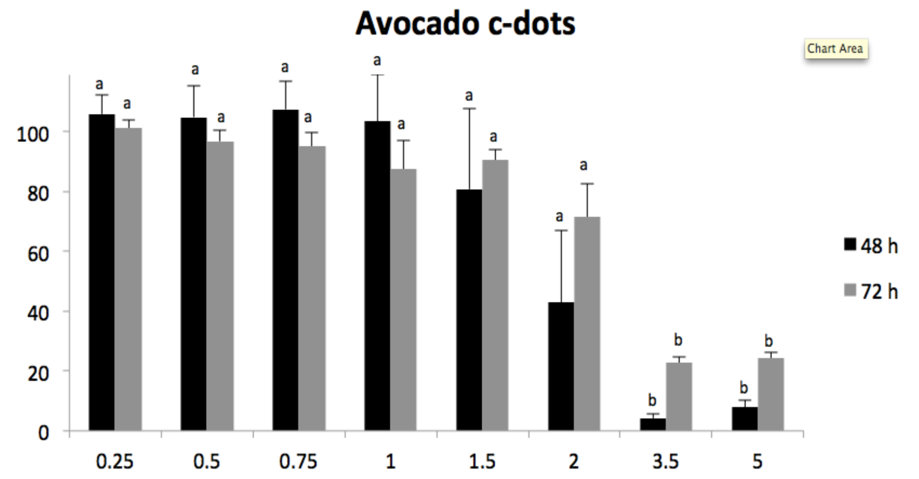

Figure S6. Caco-2 cell viability evaluation after 48 h and 72 h incubation with growing concentrations of avocado CD. Different letters indicate significant differences among treatment (P<0.05). 48 h: F(8, 26)=4.6450, P<0.05. 72 h: F(8, 27)=21.2970, P<0.05.

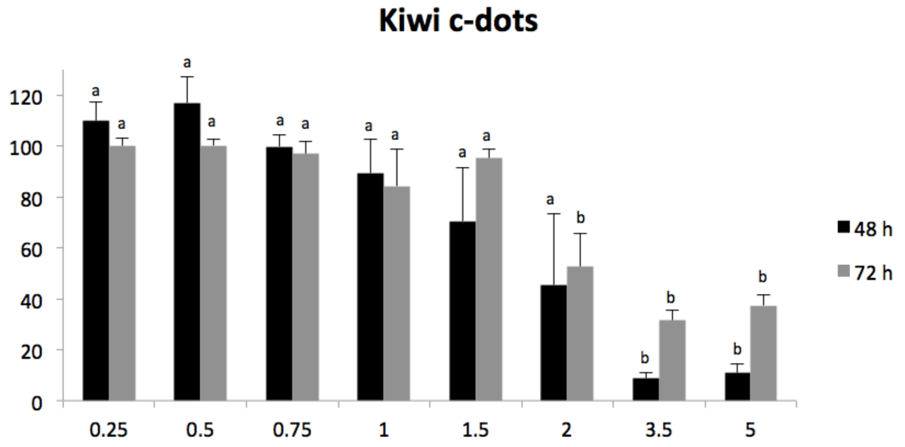

Figure S7. Caco-2 cell viability evaluation after 48 h and 72 h incubation with growing concentrations of kiwi CD. Different letters indicate significant differences among treatment (P<0.05). 48 h: $F(8, 26)$=6.0047, P<0.05. 72 h: $F(8, 27)$=12.7540, P<0.05.

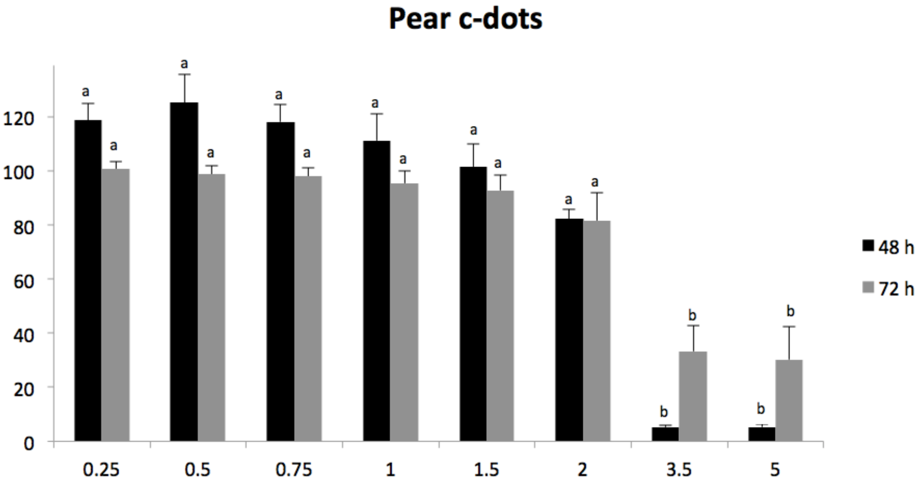

Figure S8. Caco-2 cell viability evaluation after 48 h and 72 h incubation with growing concentrations of pear CD. Different letters indicate significant differences among treatment (P<0.05). 48 h: $F(8, 26)$=16.398, P<0.05. 72 h: $F(8, 27)$=14.948, P<0.05.

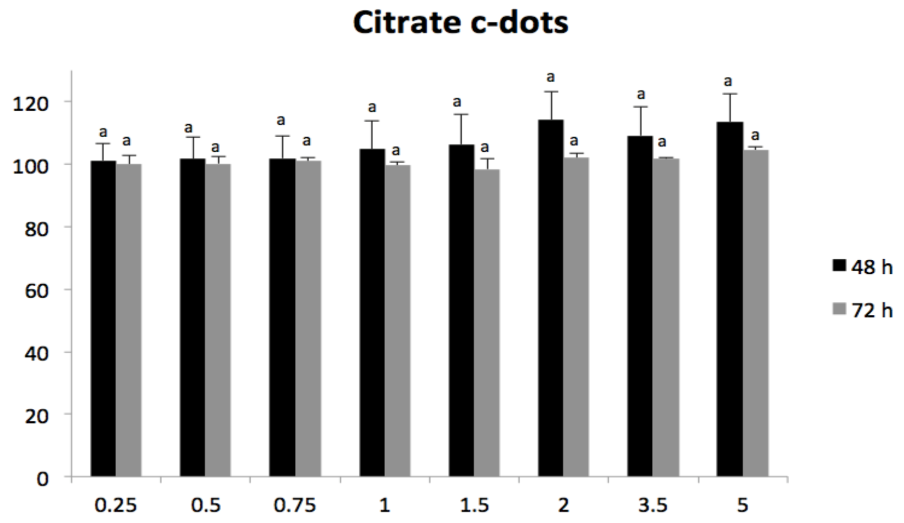

Figure S9. Caco-2 cell viability evaluation after 48 h and 72 h incubation with growing concentrations of citrate CD. Different letters indicate significant differences among treatment (P<0.05). 48 h: $F(8, 26)$=1.0935, P=0,3987. 72 h: $F(8, 27)$=0.4010, P=0,9101.

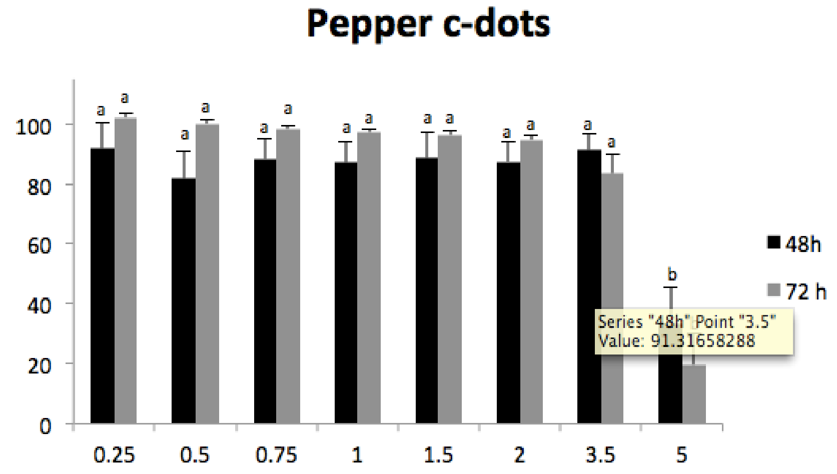

Figure S10. HK-2 cell viability evaluation after 48 h and 72 h incubation with growing concentrations of pepper CD. Different letters indicate significant differences among treatment (P<0.05). 48 h: $F(8, 42)$=8.7523, P<0.05. 72 h: $F(8, 36)$=101.1400, P<0.05.

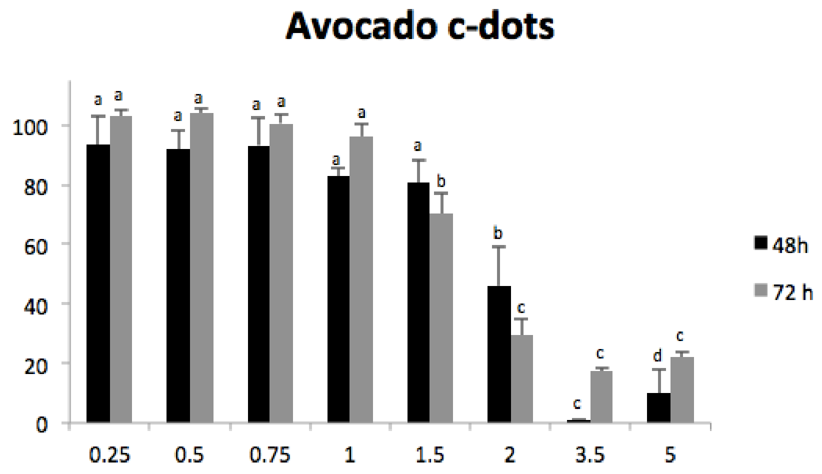

Figure S11. HK-2 cell viability evaluation after 48 h and 72 h incubation with growing concentrations of avocado CD. Different letters indicate significant differences among treatment ($P<0.05$). 48 h: $F(8, 51)=22.0340$, $P<0.05$. 72 h: $F(8, 36)=101.1400$, $P<0.05$.

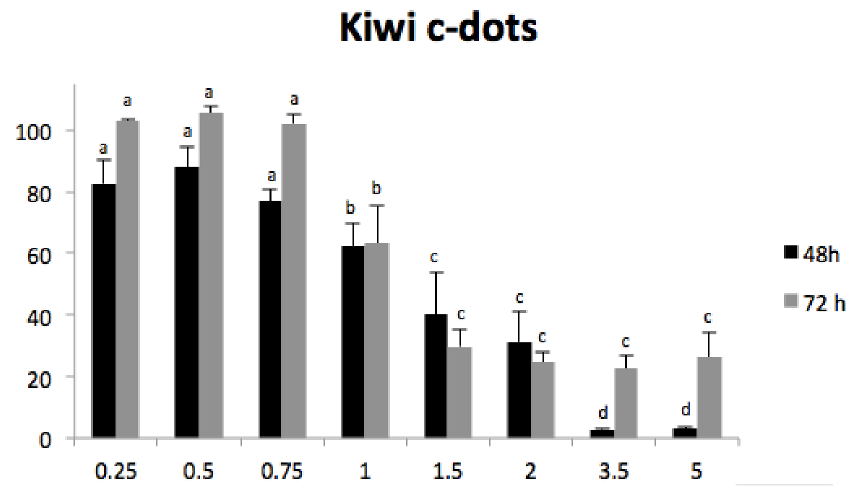

Figure S12. HK-2 cell viability evaluation after 48 h and 72 h incubation with growing concentrations of kiwi CD. Different letters indicate significant differences among treatment ($P<0.05$). 48 h: $F(8, 51)=19.615$, $P<0.05$. 72 h: $F(8, 36)=53.115$, $P<0.05$.

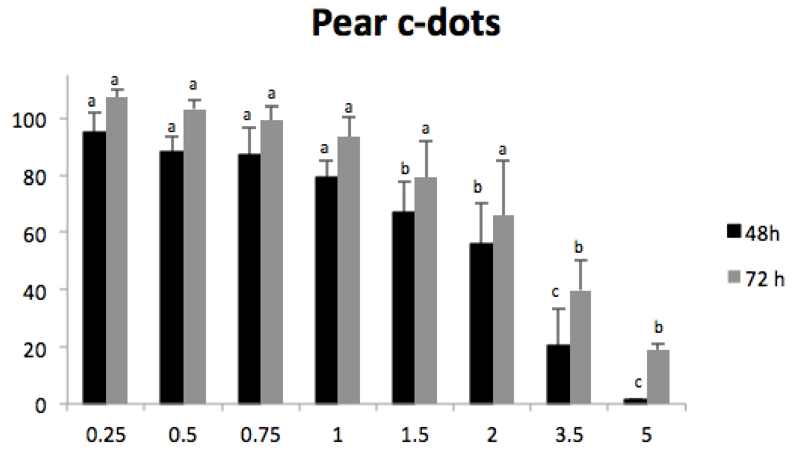

Figure S13. HK-2 cell viability evaluation after 48 h and 72 h incubation with growing concentrations of pear CD. Different letters indicate significant differences among treatment ($P<0.05$). 48 h: $F(8, 51)=18.884$, $P<0.05$. 72 h: $F(8, 36)=10.496$, $P<0.05$.

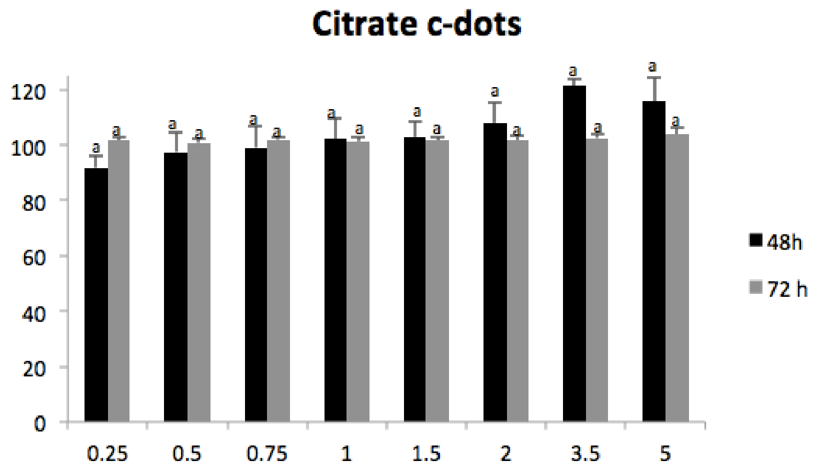

Figure S14. HK-2 cell viability evaluation after 48 h and 72 h incubation with growing concentrations of citrate CD. Different letters indicate significant differences among treatment (P<0.05). 48 h: F(8, 51)=1.5769, P=0.1551. 72 h: F(8, 36)=1.1170, P=0.

References

1. V. N. Mehta, S. Jha, H. Basu, R. K. Singhal and S. K. Kailasa, Sens. Actuators, B, 2015, 213, 434–443.
2. F. Du, M. Zhang, X. Li, J. Li, X. Jiang, Z. Li, Y. Hua, G. Shao, J. Jin, Q. Shao, M. Zhou and A. Gong, Nanotechnology, 2014, 25, 315702
3. J. Zhang, Y. Yuan, G. Liang and S.-H. Yu, Adv. Sci., 2015, 2, 1500002.
4. Vasimalai, N.; Vilas-Boas, V.; Gallo, J.; Cerqueira, M. de F.; Menéndez-Miranda, M.; Costa-Fernández, J.M.; Diéguez, L.; Espiña, B.; Fernández-Argüelles, M.T. Beilstein J. Nanotechnol. 2018, 9, 530–544.
5. Z. L. Wu, P. Zhang, M. X. Gao, C. F. Liu, W. Wang, F. Leng and C. Z. Huang, J. Mater. Chem. B, 2013, 1, 2868–2873.
6. M. Saxena and S. Sarkar, Mater. Express, 2013, 3, 201–209.

7. A.-M. Alam, B.-Y. Park, Z. K. Ghouri, M. Park and H.-Y. Kim, Green Chem., 2015, 17, 3791–3797.
8. B. S. B. Kasibabu, S. L. D'souza, S. Jha and S. K. Kailasa, J. Fluoresc., 2015, 25, 803–810.
9. P.-C. Hsu, Z.-Y. Shih, C.-H. Lee and H.-T. Chang, Green Chem., 2012, 14, 917–920.
10. C. D'Angelisdo, E. S. Barbosa, J. R. Correˆa, G. A. Medeiros,G. Barreto, K. G. Magalha˜es, A. L. de Oliveira, J. Spencer, M. O. Rodrigues and B. A. D. Neto, Chem. – Eur. J., 2015, 21, 5055–5060.
11. Pal, T.; Mohiyuddin, S.; Packirisamy, G. ACS omega 2018, 3, 831–843.
12. S. L. D'souza, B. Deshmukh, J. R. Bhamore, K. A. Rawat, N. Lenka and S. K. Kailasa, RSC Adv., 2016, 6, 12169–12179.
13. Y. Xu, D. Li, M. Liu, F. Niu, J. Liu and E. Wang, Sci. Rep., 2017, 7, 4499.
14. S. Zhao, M. Lan, X. Zhu, H. Xue, T.-W. Ng, X. Meng, C.-S.Lee, P. Wang and W. Zhang, ACS Appl. Mater. Interfaces, 2015, 7, 17054–17060.
15. C. Yang, R. Ogaki, L. Hansen, J. Kjems and B. M. Teo, RSC Adv., 2015, 5, 97836–97840.
16. C.-L. Li, C.-M. Ou, C.-C. Huang, W.-C. Wu, Y.-P. Chen, T.-E. Lin, L.-C. Ho, C.-W. Wang, C.-C. Shih, H.-C. Zhou, Y.-C. Lee, W.-F. Tzeng, T.-J. Chiou, S.-T. Chu, J. Cang and H.-T. Chang, J. Mater. Chem. B, 2014, 2, 4564–4571.
17. H. Huang, Y. Xu, C.-J. Tang, J.-R. Chen, A.-J. Wang and J.-J. Feng, New J. Chem., 2014, 38, 784–789.
18. D. Sun, R. Ban, P.-H. Zhang, G.-H. Wu, J.-R. Zhang and J.-J. Zhu, Carbon, 2013, 64, 424–434.
19. X. Yang, Y. Zhuo, S. Zhu, Y. Luo, Y. Feng and Y. Dou, Biosens. Bioelectron., 2014, 60, 292–298.
20. X. Teng, C. Ma, C. Ge, M. Yan, J. Yang, Y. Zhang, P. C. Morais and H. Bi, J. Mater. Chem. B, 2014, 2, 4631–4639.
21. H. Ding, Y. Ji, J.-S. Wei, Q.-Y. Gao, Z.-Y. Zhou and H.-M. Xiong, J. Mater. Chem. B, 2017, 5, 5272–5277.
22. M. Xue, M. Zou, J. Zhao, Z. Zhan and S. Zhao, J. Mater. Chem. B, 2015, 3, 6783–6789.
23. C. J. Jeong, A. K. Roy, S. H. Kim, J.-E. Lee, J. H. Jeong, I. In and S. Y. Park, Nanoscale, 2014, 6, 15196–15202.
24. L. Wang and H. S. Zhou, Anal. Chem., 2014, 86, 8902–8905.
25. D. Wang, X. Wang, Y. Guo, W. Liu and W. Qin, RSC Adv., 2014, 4, 51658–51665.
26. C. Phadke, A. Mewada, R. Dharmatti, M. Thakur, S. Pandey and M. Sharon, J. Fluoresc., 2015, 25, 1103–1107.
27. C. Jiang, H. Wu, X. Song, X. Ma, J. Wang and M. Tan, Talanta, 2014, 127, 68–74.
28. S. Sahu, B. Behera, T. K. Maiti and S. Mohapatra, Chem. Commun., 2012, 48, 8835–8837.
29. R. Bandi, B. R. Gangapuram, R. Dadigala, R. Eslavath, S. S. Singh and V. Guttena, RSC Adv., 2016, 6, 28633–28639.
30. K. Bankoti, A. P. Rameshbabu, S. Datta, B. Das, A. Mitra and S. Dhara, J. Mater. Chem. B, 2017, 5, 6579–6592.
31. N. Wang, Y. Wang, T. Guo, T. Yang, M. Chen and J. Wang, Biosens. Bioelectron., 2016, 85, 68–75.
32. M. Xue, Z. Zhan, M. Zou, L. Zhang and S. Zhao, New J. Chem., 2016, 40, 1698–1703.
33. X. Wen, L. Shi, G. Wen, Y. Li, C. Dong, J. Yang and S. Shuang, Sens. Actuators, B, 2016, 235, 179–187.
34. M. Tan, L. Zhang, R. Tang, X. Song, Y. Li, H. Wu, Y. Wang, G. Lv, W. Liu and X. Ma, Talanta, 2013, 115, 950–956.
35. V. N. Mehta, S. Jha, R. K. Singhal and S. K. Kailasa, New J. Chem., 2014, 38, 6152–6160.
36. V. N. Mehta, S. Jha and S. K. Kailasa, Mater. Sci. Eng., C, 2014, 38, 20–27.
37. J. Shen, S. Shang, X. Chen, D. Wang and Y. Cai, Mater. Sci. Eng., C, 2017, 76, 856–864.
38. A. Mewada, S. Pandey, S. Shinde, N. Mishra, G. Oza, M. Thakur, M. Sharon and M. Sharon, Mater. Sci. Eng., C, 2013, 33, 2914–2917.
39. S. K. Bhunia, N. Pradhan and N. R. Jana, ACS Appl. Mater. Interfaces, 2014, 6, 7672–7679.
40. Y. Hu, J. Yang, J. Tian, L. Jia and J.-S. Yu, Carbon, 2014, 77, 775–782.
41. J. Zhou, Z. Sheng, H. Han, M. Zou and C. Li, Mater. Lett., 2012, 66, 222–224.
